# Eating during immunotherapy sessions: a cross-sectional study of meal quality in patients with rheumatic diseases undergoing intravenous therapy with immunomodulators

**DOI:** 10.1007/s10067-026-08039-5

**Published:** 2026-03-25

**Authors:** Eleni C. Pardali, Arriana Gkouvi, Dimitrios G. Goulis, Christos Cholevas, Christina G. Katsiari, Dimitrios P. Bogdanos, Maria G. Grammatikopoulou

**Affiliations:** 1 Immunonutrition Unit, Department of Rheumatology and Clinical Immunology, Faculty of Medicine, School of Health Sciences, University of Thessaly, Larissa, Greece; 2 Unit of Reproductive Endocrinology, 1st Department of Obstetrics and Gynecology, Medical School, Aristotle University of Thessaloniki, Thessaloniki, Greece; 3 Department of Clinical Pharmacology, Faculty of Medicine, Aristotle University of Thessaloniki, Thessaloniki, Greece

**Keywords:** Abatacept, Belimumab, Diet, Immunonutrition, Immunotherapy, Infliximab, Nutrition, Rituximab, Tocilizumab

## Abstract

**Introduction/objectives:**

Intravenous (IV) immunotherapy sessions are common among patients with rheumatic and musculoskeletal diseases (RMDs). Although immunotherapy plays an important role in disease management, treatment-specific evidence-based nutritional guidance is currently lacking. The present cross-sectional study aimed to evaluate meal quality in patients with RMDs on the days of IV infusion immunotherapy sessions and make recommendations on how to improve dietary intake on these days, aiming to achieve ameliorated therapeutic results with less adverse events.

**Methods:**

A total of 168 outpatient visits on days of IV immunotherapy sessions were included, involving 124 patients with RMDs. Each patient provided detailed information regarding the breakfast they had consumed prior to the session and whether they had brought with them a snack to consume during treatment. The Main Meal Quality Index (MMQI) was used to evaluate meal quality of the breakfast and snack meals of each participant, ranging between 0 to 100, with greater scores indicating better meal quality.

**Results:**

Most patients (74%) reported having consumed breakfast, while only 29% had brought a snack with them for consumption during the sessions. Median MMQI score was 54.4 for breakfast and 50.0 for snack meals, respectively. Breakfast MMQI scores were positively associated with patient age (*p* = 0.040). Belimumab administration was associated with greater breakfast quality (β = 22.23, *p* = 0.003) compared to rituximab, while tocilizumab use was independently associated with lower snack quality (β = − 11.96, *p* = 0.030). Patients with systemic lupus erythematosus and axial spondyloarthritis reported consuming a breakfast of better quality.

**Conclusion:**

Although many patients consumed breakfast prior to their IV infusion immunotherapy sessions, only a small number chose to bring a snack with them. Breakfast quality appeared to be influenced by the type of immunomodulatory therapy patients were receiving. These findings highlight the need for individualized nutritional care and tailored meal guidance on the days of IV treatment, as nutrition may represent a potentially modifiable factor warranting further investigation in relation to therapeutic response and disease outcomes.

**Table:** 

**Key Points** • *On intravenous immunotherapy treatment days, patients consumed energy-dense breakfast and meal snacks, with moderate protein and high saturated fat content.* • *Meal composition was influenced by the type of immunomodulatory therapy.* • *Individualized nutrition guidance is essential, promoting high-protein, plant-based, fiber-rich, and unsaturated fat-rich meals, to support therapy and disease outcomes.*

**Supplementary Information:**

The online version contains supplementary material available at 10.1007/s10067-026-08039-5.

## Introduction

Diet and nutrition are increasingly recognized as integral components in the regulation of immune function and disease outcomes in rheumatic and musculoskeletal diseases (RMDs) [1, 2]. Beyond their role in providing energy, nutrients actively participate in immune modulation through multiple biological pathways, exerting effects both at the gastrointestinal interface and at the systemic level, a context known as immunonutrition [3].

Several immunomodulating nutrients, such as omega-3 fatty acids, vitamins E, D, and C, nucleotides, and minerals like Selenium, Zinc, and Magnesium, influence the functioning of immune cells and inflammatory processes [4]. By attenuating inflammation, targeted nutritional strategies may complement pharmacological therapies, supporting treatment effectiveness and potentially reducing therapy-related adverse effects. In parallel, the gut microbiome has emerged as a key mediator between dietary exposure and immune regulation, as dietary patterns can influence microbial diversity and metabolic activity, thereby modulating inflammatory and immune responses [5]. Greater microbial diversity, along with the intake of probiotics, has been suggested to support immune-modulating therapies [6], while dietary fiber and other microbiota-accessible carbohydrates play a central role in maintaining a beneficial gut microbiome [6, 7].

Evidence suggests that dietary patterns characterized by high intake of processed foods, added sugars, and red meat, typical components of the Western diet, may promote immune dysregulation by impairing gut barrier integrity and increasing systemic inflammation [8]. Conversely, adherence to dietary patterns rich in anti-inflammatory foods, such as the Mediterranean diet, which emphasizes on fruit, vegetables, whole grains, nuts, seeds, and fatty fish, has been associated with improved inflammatory profiles and more favorable clinical outcomes in patients with RMDs [9]. Despite the growing body of evidence supporting the role of nutrition in modulating disease outcomes, research examining the relationship between diet quality and pharmacological response remains limited. To date, only one study has reported that a high-fiber diet combined with reduced consumption of red and processed meat may improve the likelihood of responding to biologic therapies [10]. The potential role of nutrition in optimizing the therapeutic effects of intravenous (IV) infusion immunotherapy has not yet been adequately studied, particularly among patients with RMDs. Moreover, the lack of evidence-based guidelines regarding the dietary recommendations to accompany IV treatment with immunomodulators is apparent; this lack of guidance may contribute to substantial variability in patients’ dietary practices and represents a potentially modifiable factor affecting treatment response. In addition, we hypothesized that patients may lack clear guidance regarding appropriate dietary choices on the day of treatment, potentially leading to suboptimal food selections and lower meal quality. Thus, the present study aimed to assess meal quality among patients with RMDs on the day of IV immunotherapy and to provide practical, evidence-informed suggestions on dietary choices that may help support treatment response.

## Methods

### Characteristics of the patients

The present cross-sectional study included 168 visits of 124 patients with RMD diagnoses undergoing IV immunotherapy sessions, between February 2024 to February 2025, at the Department of Rheumatology and Clinical Immunology, situated at the Larissa University Hospital. The study’s protocol was approved by the Larissa University Hospital Scientific Board (30/3rd/20–02–2025).

The inclusion criteria involved (i) patients diagnosed with at least one RMD, (ii) able to communicate effortlessly in the Greek language, (iii) undergoing an IV therapy with an immunomodulator. In the present study we use the term immunotherapy to refer to IV immune-directed treatments used in rheumatology, including biologic disease-modifying antirheumatic drugs (DMARDs), cyclophosphamide and intravenous Immunoglobulin (IVIG). There were no exclusion criteria except for having a concomitant cancer diagnosis, gestation, and age younger than 18 years; all consecutive patients meeting the inclusion criteria were recruited for the study. The characteristics of the sample are presented in Table 1.

**Table 1:** Characteristics of the patients (*N* = 168)

Variable		
Sex	Women/men (*n*, %)	113 (67.0)/55 (33.0)
Age	Women/men	64.0 (54.0–73.0)†/66.0 (52.0–77.5)†
Disease	RA/SLE/vasculitis/PsA/IIM/axSpA/Sjögren disease/RPF/SSc/Sarcoidosis/EnA (*n*, %)	59 (35.0)/27 (16.0)/29 (17.0)/14 (8.3)/14 (8.3)/11 (6.5)/4 (2.4)/4 (2.4)/4 (2.4)/1 (0.6)/1 (0.6)
Drug	rituximab/infliximab/tocilizumab/belimumab/cyclophosphamide/abatacept/IVIG (*n*, %)	57 (34.0)/39 (23.4)/25 (15.0)/17 (10.0)/16 (9.5)/11 (6.5)/3 (1.8)
BMI category	Underweight/normoweight/overweight–obesity (*n*, %)	6 (3.6)/48 (29.0)/114 (68.0)
Had breakfast	Yes/no (*n*, %)	125 (74.0)/43 (26.0)
Had snack to eat	Yes/no (*n*, %)	48 (29.0)/120 (71.0)
Duration of IV session (h)		2.50 (2.0–5.0)†
Duration of IV session/categories	≤ 1 h/1–3 h/3–6 h/> 6 h	35 (21.0)/55 (33.0)/63 (38.0)/14 (8.0)

†median (IQR)

*axSpA *axial spondyloarthritis, *BMI *body mass index, *EnA *enteropathic arthritis, *h *hour, *IIM *idiopathic inflammatory myopathies, *IQR *interquartile range, *IV *Intravenous, *IVIG *intravenous immunoglobulin, *n *number, *PsA *psoriatic arthritis, *RA *rheumatoid arthritis, *RPF *retroperitoneal fibrosis, *SE *standard error, *SLE *systemic lupus erythematosus, *SSc *systemic sclerosis

For easier classification of RMD diagnoses, immunoglobulin A (IgA) vasculitis, eosinophilic granulomatosis with polyangiitis (EGPA), microscopic polyangiitis (MPA), granulomatosis with polyangiitis (GPA), and giant cell arteritis (GCA) were grouped as “vasculitis.” The sample also included patients with rheumatoid arthritis (RA), systemic lupus erythematosus (SLE), psoriatic arthritis (PsA), enteropathic arthritis (EnA) idiopathic inflammatory myopathies (IIM), axial spondyloarthritis (axSpA), Sjögren’s disease, retroperitoneal fibrosis (RPF), systemic sclerosis (SSc) and sarcoidosis.

### Data collection

All data were recorded during the IV immunotherapy sessions of each patient. The anthropometric measurements of patients were obtained according to standardized procedures by an experienced dietitian (E.C.P.). Body weight and height were measured using a digital floor scale (Kern MPE 200 K-1PEM, Kern, Germany) and a stadiometer (Seca 220, Hamburg, Germany), respectively. Body mass index (BMI) was calculated for all patients, who were subsequently classified into three categories: underweight (BMI < 18.5 kg/m^2^), normal body weight (BMI ≥ 18.5 and < 25 kg/m^2^), and overweight/obesity (BMI ≥ 25 kg/m^2^).

Patients were asked whether they had consumed breakfast on the morning of their visit and whether they had brought a snack with them during IV sessions. Detailed information on the portion sizes and composition of the breakfast and snack meals was recorded by an experienced dietitian (E.C.P.). Diet recalls were analyzed using the Athlisis dietary analysis software (Athlisis Health Development I.K.E., Athens, Greece) [11] for each meal separately.

### Main Meal Quality Index

Meal quality was assessed using the Main Meal Quality Index (MMQI), as described by Gorgulho et al*.* [12], and applied to the breakfast and snack consumed during the day of IV therapy. The MMQI is a composite dietary quality index consisting of 10 components: fruit, vegetables (excluding potatoes), animal protein-to-total protein ratio, dietary fiber, carbohydrates, total fat, saturated fat, processed meat, sugary beverages and desserts, and energy density. Each component is scored from 0 to 10 based on the individual reported intake, yielding a total score ranging between 0 and 100, with greater scores indicating better meal quality.

### Statistical analyses

Continuous variables were summarized as means ± standard deviation or medians (with the respective interquartile ranges), as appropriate, while categorical variables were presented as counts and percentages. Normality of continuous variables was assessed using the Shapiro–Wilk test and visual inspection of distributions. The Wilcoxon signed-rank test was used to compare MMQI components between breakfast and snack meals among visits where both meals were consumed. Because MMQI scores were not normally distributed and participants had repeated visits, associations between meal quality and clinical characteristics were assessed using linear mixed-effects models with patient-specific random intercepts. Linear mixed-effects models were fitted to evaluate the association between immunomodulatory treatment and MMQI scores for breakfast and snack outcomes. An analogous model was applied to examine the association between underlying disease diagnosis and MMQI scores. Each model included age, sex, BMI, and duration of IV treatment (in hours) as fixed effects, and a random intercept for participant. RMD diagnosis was excluded to avoid multicollinearity with treatment. Models were fitted using restricted maximum likelihood (REML). The overall effect of the immunomodulatory drug was tested by likelihood ratio tests comparing models with *vs*. without the drug term based on maximum likelihood (ML). Estimated marginal means (EMMs) were computed for each drug group. All analyses were performed using R Studio [version 4.5.2 (2026.01.0 + 392), R Foundation for Statistical Computing, Vienna, Austria] [13], with the significance level set at *p* < 0.05.

## Results

### Breakfast and snack consumption on the days of IV therapy

The majority of patients (74%) reported consuming breakfast on the morning of scheduled IV sessions, whereas only 29% had brought a snack with them to the hospital. The median MMQI score was higher for breakfast than for snacks among visits where both meals were consumed, without however reaching statistical significance (54.5 vs. 50.0, *p* = 0.10, *n* = 31). The median energy density of the breakfast and snack meals was 2.37 kcal/g and 2.68 kcal/g (*p* = 0.025), respectively (Supplementary Table 1). Fiber content was higher in the consumed snacks compared to the breakfast meals (*p* = 0.018). Fruit, vegetables, and sugary desserts were rarely consumed in either breakfast, or snacks. Processed meat consumption was higher in snacks than in breakfast (*p* = 0.0037) meals, as was the saturated fat content (median 14.21% in snacks vs. 10.27% in breakfast meals, *p* = 0.007). In contrast, the ratio of animal-to-total protein was higher among breakfast meals (*p* = 0.018). No significant associations were observed between the duration of IV therapy and the consumption of breakfast or snacks (data not shown).

### Associations between breakfast MMQI and clinical characteristics

Linear mixed-effects modeling was applied to the visits in which breakfast meals were consumed, with patient-specific random intercepts to account for repeated measures. After adjustment for age, sex, BMI, and duration of the IV therapy sessions, age was positively associated with breakfast MMQI scores (β = 0.27, *p* = 0.040), indicating greater meal quality among older patients.

**Fig. 1:**
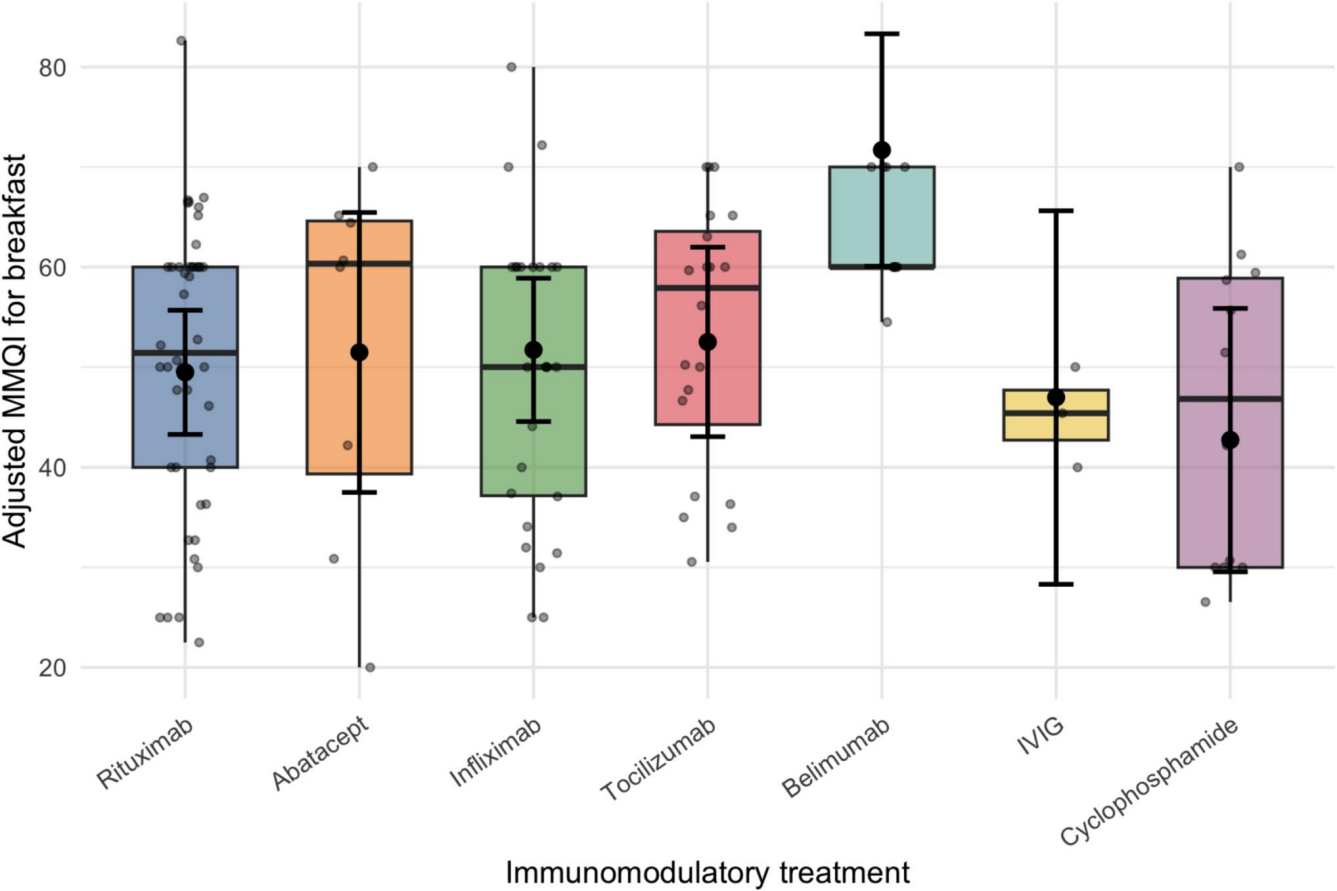
Adjusted MMQI total score by IV immunomodulatory treatment among patients who consumed breakfast. Boxplots display the distribution of MMQI total scores by infused drug, with individual data points overlaid. Grey points and error bars represent the estimated marginal means (EMMs) and 95% confidence intervals from a linear mixed model adjusted for age, sex, BMI, and duration of the therapy, with a random intercept for participant. The model tested the association between drug treatment and MMQI total score (*n* = 112). The overall drug effect was assessed by likelihood ratio test comparing models with and without immunomodulators. BMI: body mass index; IV: intravenous; IVIG: intravenous immunoglobulin; MMQI: main meal quality index

Immunomodulatory infusion was associated with breakfast MMQI, as demonstrated by a likelihood-ratio test comparing mixed-effects models with and without drug (χ^2^ = 14.7, df = 6, *p* = 0.023). In the fully adjusted model, belimumab administration was associated with greater MMQI scores (β = 22.23, *p* = 0.003), whereas other treatments (abatacept, infliximab, etc.) failed to show differences compared to the reference category (rituximab). Sex, BMI, and duration of IV therapy session were not associated with breakfast MMQI. Full model estimates are presented in Table 2. The distribution of breakfast MMQI scores across immunomodulatory treatments is detailed in Fig. 1, using boxplots with overlaid jittered individual observations.

**Table 2:** General linear model examining factors associated with breakfast MMQI among patients with RMDs on the day of IV therapy sessions (*n* = 112)

Predictor	Est	SE	*t*	*p* value
Age	0.27	0.13	2.09	0.04*
Sex (women)	−3.50	3.26	−1.08	0.29
BMI (kg/m^2^)	−0.05	0.28	−0.18	0.85
Duration of IV session (h)	0.96	1.25	0.77	0.44
Abatacept	2.01	8.40	0.24	0.81
Infliximab	2.24	5.19	0.43	0.67
Tocilizumab	3.04	6.39	0.48	0.63
Belimumab	22.23	7.28	3.05	0.0029*
IVIG	−2.50	8.81	−0.28	0.78
Cyclophosphamide	−6.76	5.73	−1.18	0.24

Reference drug is rituximab

*BMI *body mass index, *h *hour, *IV *intravenous, *IVIG *intravenous immunoglobulin, *kg *kilogram, *m *meter, *MMQI *main meal quality index, *RMDs *rheumatic and musculoskeletal diseases, *SE *standard error

The linear mixed-effects model examining associations between disease category and MMQI scores showed that SLE (β = 12.67, *p* = 0.007), and axSpA (β = 26.86, *p* = 0.03) were significantly associated with higher breakfast quality scores, while other disease diagnoses were not. The complete results of the model are presented in Supplementary Table 2.

### Associations between snack MMQI and clinical characteristics

**Fig. 2:**
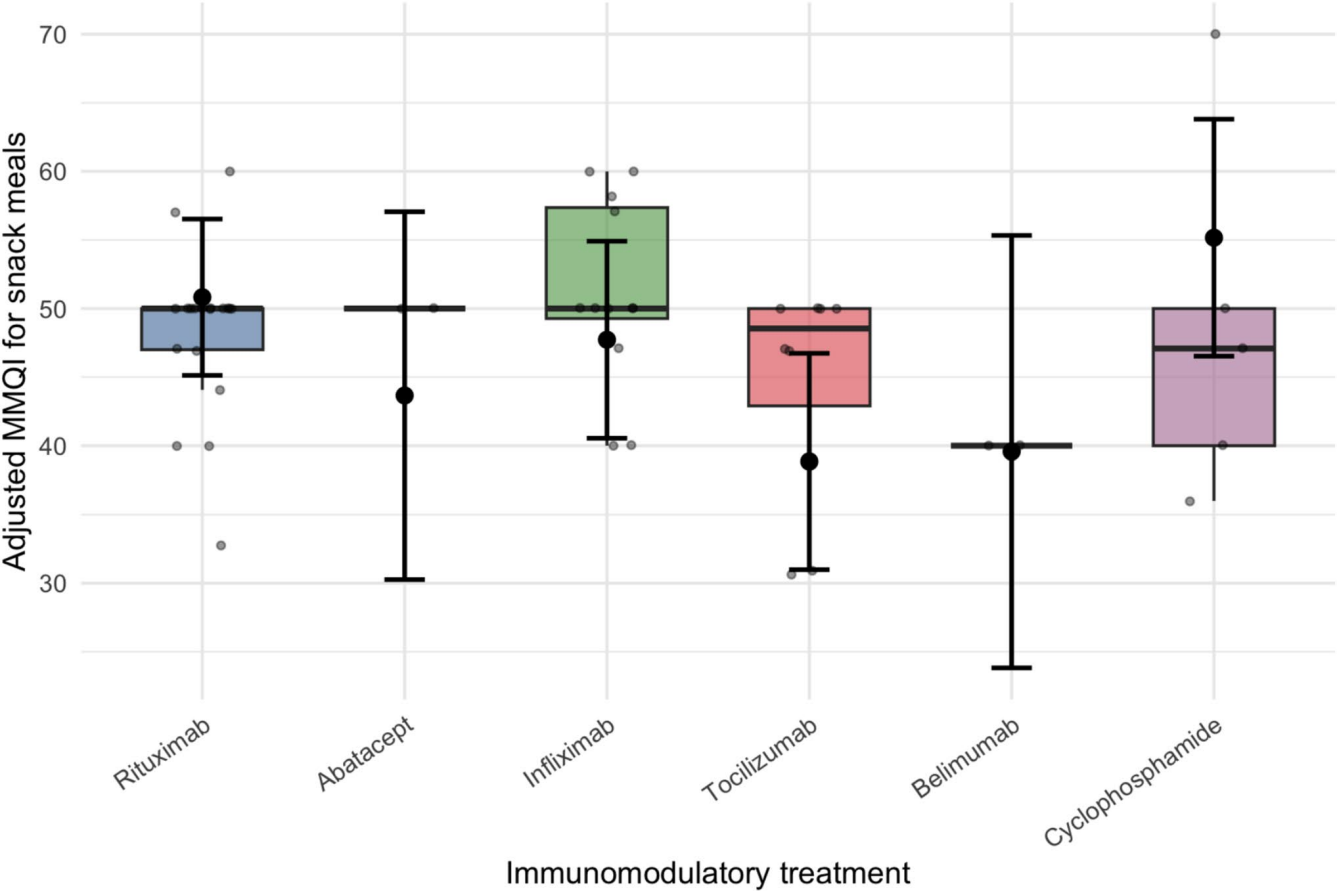
Adjusted MMQI total snack score by IV immunomodulatory treatment among patients who consumed snack meals. Boxplots display the distribution of MMQI total snack scores by administered drug, with individual data points overlaid. Grey points and error bars represent the estimated marginal means (EMMs) and 95% confidence intervals from a linear mixed model adjusted for age, sex, BMI, and duration of the therapy, with a random intercept for participant. The model tested the association between drug treatment and MMQI total snack score (*n* = 43). Although tocilizumab showed a significant negative coefficient compared to rituximab, the overall drug effect was not significant according to the results of the likelihood ratio test. BMI: body mass index; IV: Intravenous; MMQI: main meal quality index

A separate mixed-effects model was fitted for the visits in which a snack was consumed (Table 3). Due to the smaller number of observations, associations were generally weaker; however, the modeling approach remained consistent with the breakfast meal analyses. Immunomodulatory treatment was not associated with snack MMQI, as demonstrated by a likelihood-ratio test comparing mixed-effects models with and without drug (χ^2^ = 9.02, df = 5, *p* = 0.109). In the fully adjusted model, tocilizumab was associated with lower MMQI scores compared with rituximab (β = − 11.96, *p* = 0.030), while other treatments showed no statistically significant differences. Sex, BMI, and therapy duration were not associated with the quality of the consumed snack. Full model estimates are presented in Table 3. The distribution of snack MMQI scores across immunomodulatory treatments is presented in Fig. 2, using boxplots with overlaid jittered individual observations. Supplementary Table 3 details the results of the general linear model, including the disease category for snack MMQI. No significant associations were observed for disease category, age, sex, or BMI.

**Table 3:** General linear model examining factors associated with snack MMQI among patients with RMDs on the day of IV therapy sessions (*n* = 43)

Predictor	Est	SE	*t*	*p* value
Age	0.10	0.10	1.01	0.32
Sex (women)	0.78	3.10	0.25	0.80
BMI (kg/m^2^)	0.16	0.28	0.56	0.58
Duration of IV session (h)	−1.89	1.06	−1.78	0.08
Abatacept	−7.17	7.72	−0.93	0.36
Infliximab	−3.10	4.97	−0.62	0.53
Tocilizumab	−11.97	5.25	−2.23	0.03*
Belimumab	−11.25	8.91	−1.26	0.21
Cyclophosphamide	4.34	3.77	1.15	0.26

Reference drug is rituximab

*BMI *body mass index, *h *hour, *IV *intravenous, *kg *kilogram, *m *meter, *MMQI *main meal quality index, *RMDs *rheumatic and musculoskeletal diseases, *SE *standard error

## Discussion

The current study revealed that meal quality was generally suboptimal among patients with RMDs undergoing IV therapy with immunomodulators, indicating the need for improving diet quality and providing nutrition education to the patients. Older patients tended to consume a breakfast meal of better quality on the mornings of IV therapy compared to younger patients. In addition, those receiving belimumab IV exhibited greater breakfast meal quality, similar to patients with SLE and axSpA. Furthermore, only 29% of the patients herein reported bringing a snack with them during IV therapy sessions, with the overall quality of this snack being classified as “moderate”. Snack MMQI scores were inversely associated with tocilizumab treatment.

Belimumab, a monoclonal antibody (mAb) directed against B-cell activating factor (BAFF), represents an important treatment option for patients with SLE exhibiting ongoing immunological and clinical disease activity. According to the results herein, SLE diagnosis was associated with improved meal quality regarding the consumed breakfast. SLE is often associated with manifestations such as anxiety disorders, depression, and cognitive dysfunction [14, 15]. These symptoms, alongside the burden of chronic disease, can have a negative influence on the patient's dietary behaviors and eating patterns. Previous studies have suggested that patients with SLE often adhere to dietary modifications, such as favoring plant-based diets and reducing the intake of animal products and processed foods, often with the aim of alleviating disease-related symptoms [16]. Neuropsychiatric burden, body‑image issues, changes in body weight, and strong “eat healthy for lupus” messaging together create a context in which disordered and orthorexic eating behaviors can emerge, though precise prevalence and mechanisms in SLE remain under‑researched [17, 18]. Indeed, disordered eating behaviors have been reported in this population [17, 19–21], especially among patients with excessive body weight [22]. Emerging evidence suggests a tendency towards orthorexic eating behaviors among patients with SLE [23]. These orthorexic attitudes might possibly explain the improved meal quality observed among patients with SLE herein. Another possible explanation for the improved breakfast quality among patients on belimumab might be lupus nephritis. Lupus nephritis consists of a common SLE complication, affecting 25–60% of patients [24], while requiring adherence to specific dietary recommendations [25]. These dietary restrictions may be the drivers of improved meal quality among patients with lupus nephritis on belimumab.

In the present study, Tocilizumab (mAb) administration was independently associated with lower snack quality scores. While statistically significant, this finding should be interpreted with caution as it may not reflect a direct pharmacologic effect of IL-6 inhibition, but rather stem from various clinical and behavioral patient characteristics. Tocilizumab is typically used in the management of moderate-to-severe RA and GCA [26–28]. Thus, it may act as a surrogate marker of higher cumulative inflammatory burden and greater functional impairment [29]. Research suggests that Tocilizumab increases appetite, body weight and serum adipokine levels [30–32], inducing a state indicative of leptin resistance and increased hunger sensation, which may drive the selection of more palatable snacks. Tocilizumab is well recognized for its ability to suppress acute-phase reactants such as C-reactive protein (CRP), thereby potentially limiting the detection of concurrent inflammation [33, 34]. Another plausible explanation relates to glucocorticoid co-exposure, since many patients with GCA are receiving high-doses of glucocorticoids [35]. Glucocorticoids can increase appetite, enhance preference for refined carbohydrates and alter reward-related eating behaviours [36, 37]. Not capturing cumulative glucocorticoid exposure constitutes a limitation of the present study. Furthermore, there are some reports of gastrointestinal adverse events, including dyspepsia and abdominal pain [38–40] with regards to the drug [41]. Such symptoms are likely to influence patients’ eating patterns and food choices, potentially favoring foods that are easier to tolerate. In this context, patients may be more inclined to opt for foods that are palatable, easy to consume or readily available, including processed options. In addition, limited awareness of the dietary recommendations for attaining health may represent an additional barrier to making healthier food choices [42]. Consequently, maintaining adequate dietary intake while maintaining nutritional quality may be more challenging in this population. Conversely, given the smaller number of patients consuming snacks on the day of IV treatment and the non-significant drug effect, this finding should be considered exploratory.

Although our study observed greater breakfast quality on the day of IV therapy, evidence on the habitual diet of patients with axSpA is limited. Existing studies suggest an overall suboptimal dietary intake [43, 44] paired with a high intake of dietary supplements [45], highlighting an existing research gap.

Nutritional advice tailored for immunotherapy sessions (Table 4) emphasizes on the importance of maintaining adequate hydration status and avoiding overeating, as well as limiting foods that may exacerbate gastrointestinal symptoms, or increase infection risk. However, there is little guidance on what patients should actively choose to consume on IV treatment days. Patients are advised to avoid heavy, greasy, or fatty foods, spicy or acidic food items (such as lemons, tomatoes, or oranges), mold-ripened soft cheeses, unpasteurized dairy products, raw eggs, undercooked red meat, poultry, or seafood, leftover rice, and fresh sprouts [46, 47]. Also, dietary counseling with a registered dietitian is recommended primarily for patients who experience changes in appetite sensation or require guidance for managing changes in body weight [48]. Nevertheless, these recommendations remain largely generic and are not tailored to specific disease diagnoses or therapeutic regimens, while actual patient behavior highlights the need for more practical and individualized nutritional guidance during IV treatment sessions.

**Table 4:** Nutritional advice for patients undergoing IV sessions with immunomodulators

Drug	Adverse events	Foods to avoid	What to do during the infusion
Abatacept	Diarrhea, nausea, abdominal pain, dyspepsia, mouth ulceration, aphthous stomatitis, vomiting, gastritis, dizziness, fatigue [73, 74]	Mold-ripened soft cheese, unpasteurized dairy products, raw eggs, undercooked red meat, poultry, or seafood, leftover rice, fresh sprouts, unwashed products [47]	Nothing reported
Belimumab	Diarrhea, nausea [75, 76]	Nothing reported	Nothing reported
Cyclophosphamide	Nausea, vomiting, appetite loss, dizziness, light-headedness, shortness of breath, diarrhea, stomach pain, cardiotoxicity, hepatotoxicity, bladder toxicity [77, 78]	Raw food, hard and crunchy foods, acidic foods, salty foods, spicy foods, greasy foods [79], grapefruit and grapefruit juice for 48 h before and on the day of the therapy [80]	During treatment drink sufficient fluids to promote diuresis and lower the risk of urinary tract complications [81]. To minimize the chance of hemorrhagic cystitis, urine output should be maintained > 100 mL/h throughout the infusion [82]. Drink much water; dexrazoxane (1000 mg/m^2^), coenzyme Q10 (200 mg/day) selenium (100–200 μg/day), vitamin E (300 or 600 mg/day), omega-3 fatty acids (500 mg/day), berberine (10–20 mg/kg/day), vitamin D for cardiotoxicity; silymarin (milk thistle; 60 mg/kg/day), N-acetylcysteine (600 mg/day), vitamin E (300/600 mg/day), ursodeoxycholic acid (up to 20 mg/kg/day) for hepatotoxicity [83] In case of diarrhea, try eating low-fiber, bland foods, such as white rice and boiled or baked chicken [79]. Avoid raw fruits, vegetables, whole-grain breads, cereals, and seeds [79]. In case of mucositis, eat moist foods and drink plenty of fluids [79]
Infliximab	Stomach pain, indigestion/dyspepsia, vomiting, nausea, headaches, dizziness [84–86]	Interactions with food have not been established [87]	Drink much water [88]. Opt for dietary fiber from grains, fruit, and vegetables; marine n-3 PUFA; supplement with vitamin D if necessary [89]. Anthocyanins may improve the effectiveness of infliximab [90]
IVIG	Abdominal pain, anorexia, nausea, vomiting, diarrhea, headache, fatigue [91, 92]	Nothing reported	Important role of prehydration [93]
Rituximab	Diarrhea, nausea, vomiting, abdominal pain, bowel obstruction, GI perforation, GI infections [94, 95]	Heavy or greasy/fatty, spicy, or acidic foods (lemons, tomatoes, oranges), mold-ripened soft cheese, unpasteurized dairy products, raw eggs, undercooked red meat, poultry, or seafood, leftover rice, fresh sprouts, grapefruit [46, 47]	Possible role of vitamin D; nothing else reported [48]
Tocilizumab	Abdominal pain, diarrhea, nausea, mouth ulcers, weight gain, headaches, dizziness, hypokalemia, constipation, GI perforation, hypercholesterolaemia [38, 39]	Nothing reported, however, due to the risk of hypercholesterolaemia, a heart-healthy diet is advised	Nothing reported

*GI *gastrointestinal, *IBD *Inflammatory bowel disease, *IV *intravenous, *IVIG *intravenous immunoglobulin

In contrast to the existing recommendations, the present study revealed that most patients do not consume snacks during IV therapy sessions, instead preferring to wait until returning home to have lunch. In parallel, overall snack quality was suboptimal. This pattern suggests low adherence to snack-related dietary guidance during treatment sessions and highlights the need for more practical support, or clearer guidance to promote healthier eating during IV treatment sessions.

In addition, both breakfast and snack meals consumed by the patients were characterized by a high energy density. Rheumatic diseases are often characterized by fatigue, which can be one of the most disabling symptoms [49]. The observed fatigue may reflect the combined effects of systemic inflammation, disease burden, and treatment-related factors. Fatigue is a common symptom across many chronic inflammatory and immune-mediated conditions and has been associated with inflammatory cytokine activity, metabolic alterations, and increased physiological stress [50, 51]. In this context, the preference for higher-energy meals observed in our cohort may represent a compensatory behavior aimed at alleviating fatigue or meeting perceived increased energy demands.

However, while energy intake appeared elevated during IV sessions, protein intake remained suboptimal, contributing to less than 15% of the energy intake at both breakfast and snack meals. Adequate protein consumption is essential for lymphocyte proliferation, antibody synthesis, and preservation of muscle mass, which is often compromised in chronic inflammatory diseases [52, 53]. Moreover, patients receiving therapies such as cyclophosphamide or rituximab may experience increased catabolic stress or treatment-related fatigue, making sufficient protein intake critical for recovery and functional status [54, 55]. Malnutrition and cachexia can negatively affect immunotherapy efficacy and disease progression by impairing immune function, reducing treatment tolerance, and increasing the risk of adverse outcomes in oncology, although the relevance to rheumatology infusion therapies remains uncertain [56, 57].

In addition to the quantity of total protein intake, the quality of protein sources also warrants consideration, given that processed meat was consumed more frequently during the reported snack meals. Notably, a low intake of red and processed meat has been linked to improved odds of responding to biologic therapies [10]. Furthermore, saturated fat accounted for more than 10% of the energy intake of both breakfast and snack meals, indicating suboptimal fat quality of the consumed meals [58]. Replacing saturated fats with omega-3 polyunsaturated fatty acids may be beneficial, as they possess anti-inflammatory and pro-resolving properties that can modulate cytokine-driven inflammation [59]. The type of dietary fat has also been suggested to influence immunotherapy response in mechanistic animal studies, including monoclonal antibody treatment [60]. Specifically, extrapolating from studies in oncology, fat-based or ketogenic-like diets (high-fat, low-starch) are associated with longer progression-free survival, whereas high-starch diets correlate with poorer outcomes [61–63]. Conversely, a Western diet (high-fat, high-starch) has been associated with a greater risk of immune-related adverse events, indicating that not all high-fat diets exert similar immunological benefits [61].

Fiber intake of the sample was particularly low, with a median of 2.32 g being consumed at breakfast. Given the current recommendations of 25–30 g of fiber daily [64], this would correspond to approximately 5–7 g at breakfast and 3–5 g deriving from a snack, suggesting that the recorded fiber intake of participants during the early part of the day was inadequate. Indeed, recent evidence indicates that dietary regimens high in fiber may be associated with increased odds of responding to biologic therapies [10]. Dietary patterns rich in fiber provide microbiota-accessible carbohydrates and other nutrients that beneficially modulate gut microbial composition and function [6, 7, 65]. Baseline microbial diversity has been proposed as a predictor of treatment response, with increased abundance of *Akkermansia mucinifila, Ruminococcaceae, Faecalibacterium*, and *Lachnospiraceae,* and was associated with improved outcomes in patients undergoing immunotherapy [6, 61]. In parallel, *Lactobacillus reuteri* has been shown to alleviate the gastrointestinal toxicity of rituximab in animals [66], indicating that probiotics can potentially boost immunotherapy [67]. Importantly, while microbial diversity and specific *taxa* have been associated with treatment response in oncological immunotherapy, whether comparable microbiome strains predict response or symptom burden in rheumatology infusion therapies remains under investigation.

Specific nutrients may be especially relevant in maintaining immune competence and regulating inflammatory responses [3]. Vitamin D, a key regulator of immune tolerance, influences T-cell differentiation and regulatory T-cell function. Achievement of optimal 25-hydroxyvitamin D (25[OH]D) levels with vitamin D3 supplementation is also associated with improved outcomes in patients with deficient/insufficient 25(OH)D concentrations receiving rituximab-based treatment for B-cell lymphomas; on the other hand, evidence linking vitamin D repletion to improved outcomes in rituximab-treated rheumatology patients is limited [48]. Additionally, antioxidant micronutrients derived from whole foods, including vitamins C and E, and Selenium, may help mitigate oxidative stress associated with immune-mediated tissue injury, supporting musculoskeletal and systemic tissue integrity during inflammatory flares [3]. Other micronutrients such as Zinc, Iron, and Magnesium contribute to optimal immune and musculoskeletal health [4]. Zinc is essential for T-cell signaling and cytokine regulation, and Iron status influences fatigue, anemia, and pain of chronic disease [3, 4]. Higher vitamin K intake has been associated with longer progression-free survival, highlighting the potential relevance of diet in modulating outcomes of immune checkpoint inhibitor therapies [61]. Suboptimal intake of these micronutrients may thus contribute to fatigue, pain, and reduced quality of life during immunomodulatory therapy, potentially affecting treatment adherence and overall patient experience.

Patients undergoing IV immunotherapy should be encouraged to consume adequate energy and protein, paired with a high-fiber content, involving plant-rich food items. The intake of dietary fat should prioritize unsaturated sources such as olive oil, nuts, seeds, and oily fish, while intake of processed meats and saturated fats should be minimized [4, 68]. Furthermore, the use of oral nutritional supplements (ONS) enriched in omega‑3 fatty acids, arginine, glutamine, nucleotides, antioxidants, and prebiotic fiber may provide additional benefits for tampering inflammation, improving gut health, and preventing complications, particularly in malnourished patients [4]. Also, the inclusion of prebiotic fiber may increase the production of short-chain fatty acids (SCFAs) such as butyrate, which have anti-inflammatory effects and support intestinal health; however, most evidence linking SCFAs/microbiome profiles to “immunotherapy response” comes from research on cancer immunotherapy, and rheumatology-specific data remain scarce [69, 70].

The present findings indicate that there is room for improving patients’ dietary choices on the days of immunomodulatory IV sessions and highlight the need for the conduction of more research in order to clarify whether treatment-day nutrition is associated with overall health and treatment outcomes. Several ready-to-consume drinks are commercially available, aiming to provide an “immunonutrition boost” among patients in need. These contain fiber, protein, specific immunomodulatory amino acids –like arginine–, eicosapentaenoic acid (EPA), docosahexaenoic acid (DHA), vitamins and minerals acting as immunonutrients [3]. It would be interesting to ascertain if these drinks are effective in improving immunotherapy response among patients with RMDs, as in this case, they could consist of a viable solution, offered during IV immunotherapy sessions by the hospital staff. Alternatively, if effective, such drinks could be compensated by the health care systems in order to provide a horizontally adequate nutrition for patients with RMDs on the days of IV immunotherapy sessions, given that these infusions may last anything between 1–6 hours, during which time, all patients will ultimately be required to consume some food.

### Limitations and strengths of the present study

The present study has several limitations that should be considered. First, its cross-sectional design does not allow for causal inferences between meal quality and clinical outcomes. Additionally, the MMQI was developed to evaluate the quality of main meals consumed during the day, and thus it may not be fully appropriate for assessing minor eating events such as snacks. Nevertheless, in the absence of a validated tool designed for assessing meal quality on the day of IV immunotherapy, the MMQI was selected due to its multiple and comprehensive components and the ability to study energy density, saturated fat content, processed meat and sugary dessert intake, among others. Third, disease activity and other potentially important confounders such as glucocorticoid exposure, comorbidities, and socioeconomic factors were not assessed, which could influenced patient appetite and food choices. Additionally, it should be considered that the treatment allocated to each patient is closely related to the diagnosis and disease severity of each patient, thus drug-group differences may be affected by confounding factors. Due to the strong collinearity between drug type and underlying disease, and given the relatively small sample size, it was not feasible to include both variables simultaneously in the multivariable regression models without compromising model stability. Thus, the observed associations between drug group and meal quality should not be interpreted as causal and may reflect underlying disease-related differences. Also, some subgroup analyses were based on a limited number of observations—particularly those involving snack consumption. Furthermore, dietary intake was assessed on a single treatment day, which does not permit for conclusions regarding the habitual dietary patterns of patients. However, the study was intentionally designed to evaluate meal quality specifically on the day of IV immunotherapy, rather than long-term dietary behavior. Finally, the limited available evidence regarding nutritional considerations on immunotherapy session days is derived primarily from patients with cancer, as data in autoimmune rheumatic diseases remain scarce. The present study was not designed to determine whether improved nutritional choices enhance therapeutic efficacy, but rather to highlight the gap in the literature and underscore the need for the conduction of prospective studies addressing these clinically relevant questions. Despite these limitations, the present study is the first to assess meal quality of breakfast and snacks consumed on the days of IV sessions among patients with RMDs, and may serve as an initial step in expanding research on the dietary patterns of this population. Finally, ongoing studies are currently aiming to establish dietary recommendations for patients receiving IV immunomodulatory treatment, the findings of which may provide a valuable framework for future research [71, 72]. Asides from the dietary factors influencing immunoterapy [62], research has also revealed that the timing of immunotherapy infusion [92] is also of great importance for improved response and outcomes (chronotherapy). Future research should focus on combining these two aspects as a means for detecting the best infusion time and meal to accompany immunomodulator therapies in patients with RMDs [96].

## Conclusion

Food consumption during IV immunotherapy days appears to be suboptimal and should be improved. Patients with RMDs receiving IV infusion therapy reported consuming breakfast commonly, whereas snack intake was less frequent. Overall breakfast meal quality was suboptimal; however, increased breakfast quality was observed among individuals receiving belimumab IV. Snack meal quality was moderate, with lower quality being observed among patients on tocilizumab. Given the impact of nutrition on therapeutic outcomes, these findings underscore the need for individualized nutritional assessment and targeted dietary support, tailored to specific immunomodulatory regimens, especially for patients receiving highly toxic therapies.

## Supplementary Information

Below is the link to the electronic supplementary material.

Supplementary file1 (DOCX 27 KB)

## Data Availability

The datasets collected for this manuscript are accessible from the corresponding author upon reasonable request.
