# Supplementary material for: Eating during immunotherapy sessions: a cross-sectional study of meal quality in patients with rheumatic diseases undergoing intravenous therapy with immunomodulators
Source: Clin Rheumatol. 2026 Mar 25;45(6):3757–70. doi: 10.1007/s10067-026-08039-5 (PMC13249668; doi:10.1007/s10067-026-08039-5)
Supplement: Supplementary file 1 — Supplementary file1 (DOCX 27 KB) [file 10067_2026_8039_MOESM1_ESM.docx]

**Eating during immunotherapy sessions: meal quality in patients with rheumatic diseases undergoing intravenous therapy with immunomodulators**

**Journal Name: Clinical Rheumatology**

Eleni C. Pardali^1^, Arriana Gkouvi^1^, Dimitrios G. Goulis^2^, Christos Cholevas^3^, Christina G. Katsiari^1^, Dimitrios P. Bogdanos^1^, Maria G. Grammatikopoulou^1^

^1^Immunonutrition Unit, Department of Rheumatology and Clinical Immunology, Faculty of Medicine, School of Health Sciences, University of Thessaly, Larissa, Greece.

^2^Unit of Reproductive Endocrinology, 1st Department of Obstetrics and Gynecology, Medical School, Aristotle University of Thessaloniki, Thessaloniki, Greece.

^3^Department of Clinical Pharmacology, Faculty of Medicine, Aristotle University of Thessaloniki, Thessaloniki, Greece.

**Corresponding author:** Maria G. Grammatikopoulou, Immunonutrition Unit, Department of Rheumatology and Clinical Immunology, Faculty of Medicine, School of Health Sciences, University of Thessaly, Biopolis campus, Larissa, Greece; telephone +306974929290; email address: [mgrammat@uth.gr](mailto:mgrammat@uth.gr); ORCID: 0000-0003-4167-6595.

**Supplementary Table 1**. Components of the MMQI for breakfast and snack among patients with RMDs on the day of IV therapy sessions (n=31).

| MMQI components | Breakfast | Snack | p-value |
| --- | --- | --- | --- |
| Fruit (g) | 0 (0) | 0 (0) | 0.69 |
| Vegetable (g) | 0 (0) | 0 (0) | 0.14 |
| Animal protein/total protein (% ratio) | 50.0 (75.5) | 0 (65) | 0.018* |
| Fiber (g) | 2.32 (1.98) | 3.40 (2.53) | 0.018* |
| Carbohydrates (% of energy intake) | 60.12 (24.20) | 61.91 (24.56) | 0.45 |
| Total fat (% of energy intake) | 24.79 (25.03) | 25.83 (20.51) | 0.87 |
| Saturated fat (% of energy intake) | 10.27 (13.00) | 14.21 (23.21) | 0.007* |
| Energy density (kcal/g) | 2.37 (1.85) | 2.68 (0.38) | 0.025* |
| Processed meat (portions) | 0 (0) | 0 (0) | 0.0037* |
| Sugary dessert (portions) | 0 (0) | 0 (0) | 0.89 |
| Total MMQI | 54.5 (20.0) | 50.0 (3.79) | 0.10 |

g: grams; kcal: kilocalories; IV: intravenous; MMQI: main meal quality index; RMDs: rheumatic and musculoskeletal diseases.

**Supplementary Table 2**. General linear model examining factors associated with breakfast MMQI among patients with RMDs on the day of IV therapy sessions, including disease category (n=112).

| Predictor | Est. | SE | t | p value |
| --- | --- | --- | --- | --- |
| SLE | 12.67 | 4.55 | 2.78 | 0.007* |
| Vasculitis | 5.53 | 4.33 | 1.28 | 0.21 |
| Myositis | 4.36 | 5.35 | 0.82 | 0.42 |
| PsA | 5.04 | 5.47 | 0.92 | 0.36 |
| axSpA | 28.86 | 12.08 | 2.22 | 0.03* |
| Sjögren’s disease | 12.43 | 8.12 | 1.78 | 0.08 |
| RPF | 3.02 | 8.31 | 0.36 | 0.72 |
| SSc | 9.39 | 12.32 | 0.91 | 0.37 |
| Age | 0.31 | 0.12 | 2.53 | 0.01* |
| Sex (women) | -1.01 | 3.57 | -0.28 | 0.78 |
| BMI (kg/m^2^) | -0.04 | 0.29 | -0.14 | 0.89 |

axSpA: axial spondyloarthritis; BMI: body mass index; h: hour; IV: intravenous; IVIG: intravenous immunoglobulin; kg: kilogram; m: meter; MMQI: main meal quality index; PsA: psoriatic arthritis; RMDs: rheumatic and musculoskeletal diseases; PRF: retroperitoneal fibrosis SE: standard error; SLE: systemic lupus erythematosus. Reference disease is rheumatoid arthritis.

**Supplementary Table 3**. General linear model examining factors associated with snack MMQI among patients with RMDs on the day of IV therapy sessions, including disease category (n=43).

| Predictor | Est. | SE | t | p value |
| --- | --- | --- | --- | --- |
| SLE | -1.91 | 7.05 | -0.27 | 0.79 |
| Vasculitis | -6.69 | 6.23 | -1.08 | 0.30 |
| Myositis | -9.88 | 9.83 | -1.01 | 0.34 |
| PsA | 2.02 | 8.19 | 0.25 | 0.81 |
| Sjögren’s disease | 7.09 | 8.08 | 0.88 | 0.40 |
| Age | 0.23 | 0.17 | 1.37 | 0.19 |
| Sex (women) | 0.12 | 4.31 | 0.03 | 0.98 |
| BMI (kg/m^2^) | -0.05 | 0.61 | -0.08 | 0.94 |

BMI: body mass index; h: hour; IV: intravenous; IVIG: intravenous immunoglobulin; kg: kilogram; m: meter; MMQI: main meal quality index; PsA: psoriatic arthritis; RMDs: rheumatic and musculoskeletal diseases; SE: standard error; SLE: systemic lupus erythematosus. Reference disease is rheumatoid arthritis.
